# Radiographs and low field MRI (0.2T) as predictors of efficacy in a weight loss trial in obese women with knee osteoarthritis

**DOI:** 10.1186/1471-2474-12-56

**Published:** 2011-02-28

**Authors:** Henrik Gudbergsen, Mikael Boesen, Robin Christensen, Arne Astrup, Henning Bliddal

**Affiliations:** 1 The Parker Institute, Copenhagen University Hospital, Frederiksberg, Denmark; 2 Department of Radiology, Copenhagen University Hospital, Frederiksberg Denmark; 3 Department of Human Nutrition, Faculty of Life Science University of Copenhagen, Denmark

## Abstract

**Background:**

To study the predictive value of baseline radiographs and low-field (0.2T) MRI scans for the symptomatic outcome of clinically significant weight loss in obese patients with knee osteoarthritis.

**Methods:**

In this study we hypothesize that imaging variables assessed with radiographs and MRI scans pre-treatment can predict the symptomatic changes following a recommended clinically significant weight reduction Patients were recruited from the Department of Rheumatology, Frederiksberg Hospital, Denmark. Eligibility criteria were: age >18 years; primary osteoarthritis according to ACR; BMI > 28 kg/m2; motivation for weight loss. Subjects were randomly assigned to either intervention by low-energy diet (LED) for 8 weeks followed by another 24 weeks of dietary instruction or control-group. MRI scans and radiographs were scored for structural changes and these parameters were examined as independent predictors of changes in osteoarthritis symptoms after 32 weeks. The outcome assessor and statistician were blinded to group allocation.

**Results:**

No significant correlations were found between imaging variables and changes in Western Ontario and McMaster Universities Index of Osteoarthritis (Spearman's test, r < 0.33 and P > 0.07).

Only the LED group achieved a weight loss, with a mean difference of 16.3 kg (95%CI: 13.4-19.2;P < 0.0001) compared to the control group. The total WOMAC index showed a significant difference favouring LED, with a group mean difference of - 321.3 mm (95%CI: -577.5 to -65.1 mm; P = 0.01). No significant adverse events were reported.

**Conclusion:**

Stage of joint destruction, assessed on either radiographs or low-field MRI (0.2T), does not preclude a symptoms relief following a clinically relevant weight loss in elderly obese female patients with knee osteoarthritis.

## Background

Knee osteoarthritis (KOA) is a multi factorial disease characterized by joint-stiffness, pain and loss of function [1]. With an increasing prevalence of elderly and obese citizens, the problems of KOA is likely to escalate in the future [2-4], and as new potential treatments arise, there is a need to examine MRI evaluated structural changes in clinical trials.

A drug/treatment that can efficiently halt the degenerative nature of KOA (DMOAD) has not yet been presented, but in obese KOA patients recent studies have shown a direct relationship between weight loss and the level of symptomatic improvement [5]. This result supports earlier epidemiological findings that weight loss reduces the risk of development and progression of KOA, and that KOA related symptoms tend to worsen in obese patients [6-9]. As a consequence, overweight KOA patients are now recommended to commence weight reduction as a first line therapy [3,4]. Which diet to choose is still debated as there is no evidence to support one diet composition over others; the single most important factor is to establish a continuous energy deficit [10].

Conventional radiography is the simplest and least expensive imaging method for assessing KOA, and the K/L score remains the most widely applied system when diagnosing KOA [11,12] in clinical trials. Both low- and high field MRI provides additional information to radiographs, as these modalities have a unique ability to image all knee joint related structures [13].

The MRI modality withholds a possibility for semi-quantitative scoring of synovial thickening, joint effusion, bone marrow lesions (BMLs) and cartilage abnormalities. These structures are essential because the synovium, joint capsule and subchondral bone are highly innervated and appear to represent some of the main origins of KOA-related pain, whereas the cartilage status is suggested to be more a marker of joint strain and thereby a surrogate marker for KOA symptoms [14]. All of these structural changes have been shown to correlate with clinical symptoms and/or progression of disease [15-20], and they therefore seem relevant to examine in this intervention study.

In this study we hypothesize that imaging variables assessed with radiographs and MRI scans pre-treatment can predict the symptomatic changes following a recommended clinically significant weight reduction [5].

## Methods

### Participants

Following approval from the local ethical committee ((KF) 01-104/02 and 11-149/03), female patients were recruited from the outpatients' clinic, Department of Rheumatology, Frederiksberg Hospital, Denmark. They were all invited from the waiting list of the first diet study from the Parker Institute (Christensen, 2005 86/id). All patients signed and approved the informed consent and standing knee radiographs, MRI and clinical examinations were performed on the same day at baseline. The study was carried out in accordance with the Helsinki Declaration II and the European Guidelines for Good Clinical Practise.

Eligibility criteria were: age above eighteen years; primary KOA diagnosed according to the clinical classification of KOA [21]; no history or active presence of other rheumatic diseases that might be responsible for secondary KOA; no substantial abnormalities in haematological, hepatic, renal, cardiac or endocrine functions (including diabetes mellitus); body-mass index (BMI) ≥28 kg/m^2^, expression of a clear, unequivocal motivation for weight loss; fluent in Danish language.

Only pain medication was monitored in our project: All participants were asked not to change the previous medications for pain, i.e. maintain the same medication at same dosage. The GP was informed of the project and asked to monitor other medications, including antidiabetics.

### Imaging acquisition

Baseline MRI was obtained of a single knee, using a dedicated extremity scanner (E-Saote E-scanner, 0.2 Tesla, Software release 9.6B). In case of bilateral symptoms we examined the most symptomatic one All MRI scans were performed in the same department of radiology by a team of two radiographers applying a standardized technique. Knees were placed in a receive-only cylinder coil.

The imaging protocol used was:

A gradient echo scout followed by a saggital STIR with 4 mm slices (TR 1460, TE 24, FOV 160 × 160, matrix 256 × 256, acquisition time 5 min 10 s). Two successive T1-weighted 3 D gradient-echo sequences were acquired in the axial and saggital plane with respectively 104 and 52 adjoining 1.4 mm thick slices (TR 60 ms, TE 24 ms, 45° flip angle, field of view 150 mm, matrix 192 × 160 and voxel size 0.78 × 1.07 × 1.4 mm ^3^, acquisition time 6 min). Coronal T1-weighted spin-echo with 15 contiguous 4 mm thick slices (TR 520 ms, TE 15 ms, field of view 160 mm, matrix 192 × 160 mm, acquisition time 3 min 20 s with two signals acquired). Finally a saggital T2*-weighted two-dimensional gradient-echo sequence was acquired with 25 contiguous 4 mm thick sections (TR 60 ms, TE 24 ms, 45° flip angle, field of view 160 mm, matrix 192 × 160, acquisition time 4 min 50 s).

Bi-plane weight-bearing semi-flexed radiographs were taken of the index knee; one in the posteroanterior and one in the lateral view (in case of bilateral symptoms we used the most symptomatic knee). They were obtained at inclusion/baseline, using a Philips Optimus apparatus, and the same radiographers using a standardized protocol carried out all examinations. The ionizing radiation dose per examination was 0.006 mSv corresponding to 0.2% of the annual background radiation on earth (average background dose for humans are 2.4 mSv annually.

### Imaging evaluation

MRI scans were scored separately for four structural parameters and summed as a "Total MRI Score" to see if this construct would perform better as an imaging biomarker. Cartilage abnormalities, BMLs and synovitis were scored for the medial, lateral and patellofemoral chamber and effusion was graded according to the total amount.

Cartilage abnormalities were assessed using the T2* and the 3 D T1 weighted Gradient echo sequences. These abnormalities were graded 0-4 according to the description by Ding et al. [22], and the specific grades were as follows; grade 0, normal cartilage; grade 1, focal blistering and an intra-cartilaginous area of low signal intensity with an intact surface; grade 2, irregularities on the surface or bottom and a < 50% loss of thickness; grade 3, deep ulceration, with a > 50% loss of thickness; grade 4, full-thickness chondral wear, with exposure of the subchondral bone. BMLs were defined as poorly marginated areas of increased signal intensity in the subchondral bone on the STIR images, and they were scored according to the description by Torres et al. [23]. The grades were defined as follows; grade 0, normal; grade 1, < 25% of the chamber, grade 2, 25-50% of the chamber and grade 3, > 50% of the chamber. The degree of synovitis was scored according to Rhodes et al. on a scale ranging from 0-3 where 0 = normal, 1 = diffuse, even thickening, 2 = nodular thickening and 3 = gross nodular thickening [24]. The amount of effusion was graded from 0-3 where 0 = physiological amount, 1 = small amount, in the retropatellar space, 2 = moderate amount, slight convexity of the suprapatellar bursa and 3 = large, capsular distension with bulging of the extensor retinaculum [17,25].

Maximum global score was 12 for cartilage abnormalities and 9 for BMLs, effusion and synovitis. Minimum score was 0 for all assessed structural parameters.

The radiographs analysed using the Kellgren Lawrence scoring method (K/L), as this is a recommended and reliable method for baseline assessments of KOA using the fixed flexion protocol with antero-posterior and lateral radiographs [11]. One experienced investigator (MB), who was blinded to randomization, analyzed all radiographs and MRI scans in a random order.

### Interventions

Subjects were randomly assigned to either a control group or an intervention group who was treated with a dietary regime. This consisted of a low-energy diet (LED) for eight weeks followed by 24 weeks of conventional hypo-energetic and high protein diet. As previously described [5] the intervention-diet consisted of nutrition powder (Speasy, Dansk Droge A/S) dissolved in water and it was taken as six daily meals, giving the patient 3.4 MJ/day. This fulfilled the recommendations of daily intake of high quality protein [26]; 37 energy percent (E%) from soy protein providing the essential amino acids, 47 E% from carbohydrate, 16 E% from vegetable fat (primarily from rapeseed oil), and fibres from oat-bran (15 g/day). The LED group received nutritional instruction and behavioural therapy by an experienced dietician at weekly sessions (1.5 h/week) throughout the eight weeks. This was done to reinforce and continuously stimulate the patients' intention to loose weight, and to promote a high degree of compliance.

Patients in the control group attended a thorough two-hour session at baseline (by the same dietician who treated the LED group). The patients were given nutritional advice and recommended ordinary foods in amounts that would provide the patients with approximately 5 MJ/day. After this initial session all the patients in the control group received ideas for a diet plan in a booklet providing the participants with a variety of 'good-advices' when trying to reduce body weight. Finally the subjects in the control group were put on a waiting list for later recall to a similar dietary plan as in the intervention group. The follow-up visit was at t = 32 weeks.

### Biometric examinations

At baseline and after half a year (t = 32 weeks) the body weights (without coats, shoes etc.) of all patients' were recorded on a decimal scale (TANITA BW-800, 'Frederiksberg Vægtfabrik', Copenhagen, Denmark).

### Symptom assessment

The patient important outcome being their experience of KOA symptoms were assessed by the Western Ontario and McMaster Universities' (WOMAC) OA index, a validated disease-specific questionnaire comprised of three self-reported items; five pain-related questions of each 100 mm VAS (500 mm VAS in total); seventeen disability-related questions of each 100 mm VAS (1700 mm VAS in total); two stiffness-related questions of each 100 mm VAS (200 mm VAS in total). The patients mark their present level of symptoms, within each of the above described items, by placing a vertical line on a 100 mm horizontal line. The total WOMAC is a measure of the global KOA level of symptoms; 0 mm WOMAC representing no disease, and 2400 mm WOMAC representing worst possible state of disease [27]. This was done at baseline and again at follow-up (t = 32 weeks).

### Randomization, allocation concealment, implementation and blinding

A method of restricted randomization called minimization was used with stratifying patients according to (*i*) gender, (*ii*) BMI and (*iii*) age. This was done for every sixteen patients included, and ensured homogeneity between the groups [28].

Each randomization list was drawn up by the statistician and given to the secretariat. In order to implement the random allocation, the sequence was concealed until interventions were assigned: The secretariat informed the patients about when to meet with the dietician (i.e. only implicitly referring to group allocation). The code was not revealed to the researchers before data collection, imaging assessments and laboratory analyses were complete.

The statistician and the assessor of radiographs and MRI scans were blinded.

### Statistical methods

Clinical outcomes were analyzed as differences from baseline values (*x*_32 _- *x*_0_), and weight loss (kg) was also analyzed as a relative measure, being the percentage change from baseline ((*x*_32 _- *x*_0_)/*x*_0 _*100%). We performed a distribution-free Spearman's test of rank correlation when examining the possible relationship between imaging variables and clinical outcomes of the dietary interventions. Further analyses on significant results were carried out according to the data type. The Spearman correlation coefficient was interpreted as follows: < 0.3: none; 0.31-0.5: weak; 0.51-0.7: strong; 0.71-0.9: very strong and > 0.9: excellent. A P-value less than 0.05 (two-tailed) or a 95% confidence interval (CI) not including the null hypothesis was regarded as statistically significant. All the analyses were performed on SAS version 9.1 for Windows (Chicago, IL, USA).

## Results

### Population characteristics

**Figure 1:**
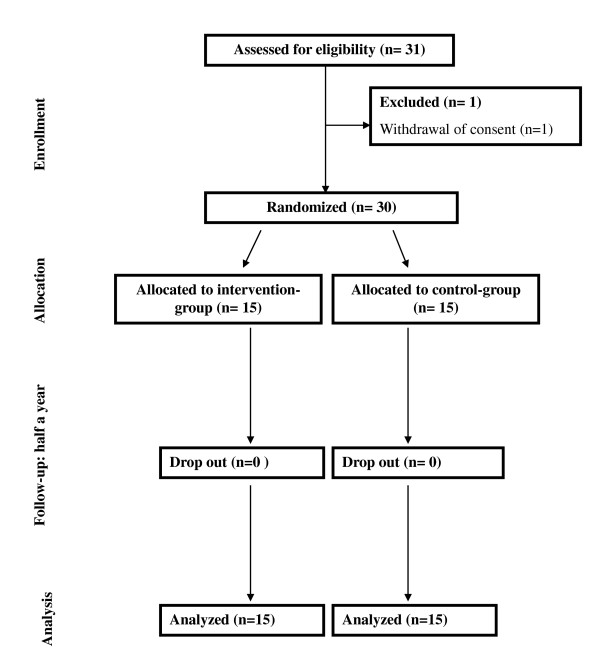
**Trial profile**.

32 patients were invited to participate, 31 of these were interested and 30 patients had baseline measurements performed. The patient not randomized was excluded due to withdrawal of consent before the randomization procedure. The 30 enrolled patients were randomly assigned to either LED or conventional hypo energetic diet. Following randomization of 15 patients to each group, all patients completed the trial and we subsequently analyzed the ITT population based on these 30 patients (Figure 1).

All patients were women, average age was 62 years (SD 6.8) and average BMI was 37 kg/m ^2 ^(SD 6.0) (see table 1). At baseline we registered data regarding blood analyses and Patient-Reported Outcomes (PROs); there were no statistically significant differences between patients in the LED and control group at baseline (data not shown). Use of pain medication was also monitored by PROs and data revealed an unaltered use during the trial period.

**Table 1:** Characteristics of all participants

	*Mean (SD) (range)* Total (n = 30)
Age (years)	62.4 (6.8) (51.2-79.6)

Height (m)	1.6 (0.06) (1.52-1.76)

Weight (kg)	99.4 (14.5) (79.1-144.8)

BMI (kg/m^2^)	36.8 (6.0) (29.4-57.3)

Sedimentation rate ^2^	16.0 (12.0; 23.0) (5.0-48.0)

C-reactive protein ^2^	5.0 (2.6; 7.5) (1.0-12.6)

Pain (mm)	197.6 (96.0) (26.0-344.0)

Disability (mm)	660.2 (345.6) (58.0-1365.0)

Stiffness (mm)	88.8 (46.5) (0.0-170.0)

Total index (mm)	944.7 (438.2) (205.0-1866.0)

	Median (range) Total (n = 30)

**Kellgren Lawrence Score**	

Medial	2 (0-4)

Lateral	1 (0-3)

Patellofemoral	2 (0-4)

Total	5 (0-9)

**MRI score**	

BML	2 (0-2)

Cartilage	4 (0-7)

Effusion	3 (0-9)

Synovitis	3 (0-6)

Total score	9 (0-20)

^1 ^Sum of visual analogue scale scores; WOMAC-pain-index of 500 mm, -disability-index of 1700 mm, -stiffness-index of 200 mm and -total-index of 2400 mm (se text in article under *Symptom assessment*)

^2 ^Showed a Non-Gaussian distribution, thus presented as median (interquartile range)

Radiographs were scored using the K/L score, and the three joint compartments were scores separately in order to assess whether KOA at specific locations had any influence on our hypothesis. No group differences (data not shown). 63% of the patients had a medial K/L score ≥ 2 and 13% had a K/L score of 0 (no group differences). The assessment of MRI scans revealed that 37% of the patients had a BML score ≥ 1 for all three compartments and that 93% of the patients had some degree of cartilage abnormalities (score ≥ 1). For effusion and synovitis 30 and 40% had a score of zero respectively.

### Assessed imaging variables as predictors of symptomatic changes following weight loss

Imaging variables as predictors of symptomatic outcome were examined by a Spearman correlation analysis (Table 2). The analysis did not show significant correlation between any imaging variables and the following outcomes; Δ WOMAC pain (mm) and Δ WOMAC disability (mm) (r ≤ 0.33; p > 0.05).

**Table 2:** Correlation of baseline imaging variables with change in symptoms

	All patients (n = 30)		The weight loss arm (n = 15)	
	**Δ Pain (%) ^1^**	**Δ Disability (%) ^1^**	**Δ Pain (%) ^1^**	**Δ Disability (%) ^1^**

*K/L score*				
Medial chamber	*r *= 0.03; (*P *= 0.86)	*r *= 0.16; (P = 0.39)	*r *= -0.15; (P = 0.59)	*r *= -0.09; (P = 0.75)
Lateral chamber	*r *= 0.10; (*P *= 0.59)	*r *= -0.03; (*P *= 0.89)	*r *= 0.14; (P = 0.61)	*r *= 0.20; (P = 0.48)
Patellofemoral chamber	*r *= 0.03; (*P *= 0.87)	*r *= -0.09; (*P *= 0.61)	*r *= -0.17; (P = 0.55)	*r *= -0.19; (P = 0.50)
Total score	*r *= 0.13; (*P *= 0.50)	*r *= -0.003; (*P *= 0.98)	*r *= -0.08; (P = 0.79)	*r *= -0.07; (P = 0.79)

*MRI score*				
BML	*r *= 0.03; (*P *= 0.86)	*r *= -0.05; (*P *= 0.80)	*r *= 0.29; (P = 0.29)	*r *= 0.03; (P = 0.91)
Cartilage	*r *= 0.10; (*P *= 0.58)	*r *= -0.09; (*P *= 0.65)	*r *= 0.04; (P = 0.90)	*r *= -0.43; (P = 0.11)
Effusion	*r *= 0.15; (*P *= 0.44)	*r *= 0.04; (*P *= 0.82)	*r *= -0.11; (P = 0.71)	*r *= 0.07; (P = 0.79)
Synovitis	*r *= 0.17; (*P *= 0.36)	*r *= 0.11; (*P *= 0.55)	*r *= -0.004; (P = 0.99)	*r *= -0.15; (P = 0.60)
Total	*r *= 0.19; (*P *= 0.31)	*r *= 0.03; (*P *= 0.88)	*r *= -0.02; (P = 0.94)	*r *= -0.18; (P = 0.52)

^1 ^Measured by the WOMAC-index

### Results regarding the weight loss program

Results from the intention-to-treat population are displayed in Table 3. The LED and control group changed their mean body weight (SE) by -15.6 (3.6)% and 0.4 (3.2)% respectively (data not shown).

**Table 3:** Results based on changes for the whole intention-to-treat population

Characteristics	*Mean (SD)* Total (n = 30)
Δ Weight (kg)	-7.8 (9.1)
Δ BMI (kg/m^2^)	-2.9 (3.4)

WOMAC index ^1^	
Δ Pain (mm)	-39 (94)
Δ Disability (mm)	-105 (299)
Δ Stiffness (m,)	-21 (42)
Δ Total index (mm)	-163 (374)

^1 ^Sum of visual analogue scale scores

In terms of responders, 40% vs. 13% of the patients in the LED and control group, respectively, achieved a pain reduction of more than 50% in the WOMAC-pain index and 33% vs. 7% of the patients in the LED and control group achieved > 50% in the WOMAC total index.. The WOMAC disability index showed improvement in the LED group when compared with the control group, MD of - 266 mm (95%CI: -468.9 to -63.1; p < 0.01) (data not shown).

### Adverse events

No significant adverse events were reported.

## Discussion

We found that KOA related structural changes seen on radiographs and MRI scans, at baseline, did not rule out improvement of symptoms following a clinically significant weight loss and could not predict the symptomatic outcome of the diet intervention in this elderly sample of female obese KOA patients. This result was found in an intervention group in which 90% of the patients experienced a significant weight reduction (> 10%), and 33% of the patients experienced > 50% reduction in their overall symptoms of KOA. The results correspond to prior studies investigating short-term effects of weight-loss and long-term outcome of total knee joint replacement [5,29]. We believe that these findings could be valuable for the future design of trials examining the benefit of weight loss in KOA patients, as it indicates that none of the examined structural parameters, individually or combined, could predict the symptomatic outcome of a significant weight loss in obese women with KOA.

A prior study investigating synovitis at baseline and clinical symptoms after two months, found no association [30], while Hill et al found a change in synovitis to be associated with change in symptoms of pain [18]. Furthermore, several cross-sectional studies have investigated MRI assessed items in relation to e.g. clinical symptoms, and in a meta-analysis BMLs and effusion/synovitis were found to be associated with knee pain [31].

The study has several limitations. It includes only 30 patients, secondly, the use of radiographs and low-field MRI are not the most advanced diagnostic tools regarding imaging assessment of osteoarthritis. Also, a follow-up period of 32 weeks might influence our findings.

The main disadvantage of low field MRI is the poorer image quality due to low SNR, which can only be compensated for by increasing either number of excitations, slice thickness and/or Field of Window or by reducing matrix and/or receiver bandwidth. All of which will increase scan time and/or decrease the in-plane resolution. Smaller cartilage abnormalities is not as well detected by low field MRI when compared to medium or high field MRI [32], but unfortunately a recent review that could have brought new insight to the subject, could not complete a meta-analysis due to study heterogeneity [33]. We applied a near isotropic sub millimetre 3 D GRE sequence and assessed images in several planes in order to achieve the highest possible diagnostic accuracy [34,35].

Finally we did not include analysis of multiple different scoring methods for radiographs and MRI scans, but the current approach was chosen inspired by several previous publications investigating this topic [17,22-25,36]. Newest evidence supports this approach as BMLs, synovitis and effusion seems to be the most important MRI assessed items likely to be associated with knee pain in KOA [31].

## Conclusion

In conclusion the present study reveals that baseline joint status assessed by low field MRI scans (0.2 T) and bi-plane standing radiographs did not influence the long-term improvement in WOMAC disability and WOMAC total indexes following a clinically relevant weight loss. The present study also demonstrated that an initial diet intervention program was able to induce a sustainable weight loss in KOA patients over a period of half a year (32 weeks).

## Competing intersts

The authors declare that they have no competing interests.

## Authors' contributions

HRG made all the analysis and interpretation of data, drafted the manuscript and approved the final version. MB contributed to the conception and design, analysed all MRI and radiographs, revised the manuscript several times and approved the final version. RC contributed to the conception and design, especially the statistics, revised the manuscript and approved the final version. AA contributed with the overall design idea, revised the manuscript and approved the final version. HB contributed to the conception and design, revised the manuscript and approved the final version.

## Pre-publication history

The pre-publication history for this paper can be accessed here:

http://www.biomedcentral.com/1471-2474/12/56/prepub
